# Simultaneous gut colonization by *Klebsiella grimontii* and *Escherichia coli* co-possessing the *bla*_KPC-3_-carrying pQil plasmid

**DOI:** 10.1007/s10096-022-04462-z

**Published:** 2022-05-28

**Authors:** Edgar I. Campos-Madueno, Carola Mauri, Elisa Meroni, Pablo Porragas Paseiro, Alessandra Consonni, Francesco Luzzaro, Andrea Endimiani

**Affiliations:** 1 Institute for Infectious Diseases (IFIK), University of Bern, Friedbühlstrasse 51, CH-3001 Bern, Switzerland; 2 Graduate School of Cellular and Biomedical Sciences, University of Bern, Bern, Switzerland; 3 Clinical Microbiology and Virology Unit, A. Manzoni Hospital, Lecco, Italy

**Keywords:** KPC, Carbapenemase, *K. oxytoca*, *K. grimontii*, pQil, Plasmid, Conjugation

## Abstract

Only two plasmid-mediated carbapenemases (KPC-2 and VIM-1) are reported in *Klebsiella grimontii*. Here, we report two *bla*_KPC-3_-positive isolates that were identified as *K. oxytoca* and *E. coli* by MALDI-TOF MS in the same rectal swab. Whole-genome sequencing indicated that *K. oxytoca* was actually *K. grimontii* of ST391, whereas *E. coli* was of ST10. In both, *bla*_KPC-3_ was carried by a pQil conjugative plasmid. The core-genome analysis identified additional *bla*_KPC_-positive *K. grimontii* strains from public databases, most of which were misidentified as *K. oxytoca*. Since *K. grimontii* represents an emerging reservoir of resistance traits, routine tools should improve their ability to detect this species.

**Supplementary Information:**

The online version contains supplementary material available at 10.1007/s10096-022-04462-z.

*Klebsiella grimontii* is an emerging pathogen associated with human infections and gut colonization that is frequently misidentified as *Klebsiella oxytoca* (e.g., implementing the matrix-assisted laser desorption ionization time of flight mass spectrometry, MALDI-TOF MS) [1, 2]. *K. grimontii* possesses a specific chromosomal β-lactamase gene (*bla*_OXY-6_) [3], but it can also acquire other antibiotic resistance genes (ARGs) via mobile genetic elements (MGEs). In particular, the recent reports of carbapenemase-producing *K. grimontii* possessing plasmid-mediated *bla*_KPC-2_ (China) and *bla*_VIM-1_ (Switzerland) are worrisome [2, 4]. Notably, very little is known about the *K. grimontii* ability to horizontally transfer such plasmids to other Enterobacterales.

In August 2020, following multiple hospitalizations caused by respiratory infections (starting in January with a respiratory syncytial virus bronchiolitis and including both methicillin-susceptible *Staphylococcus aureus* and *Haemophilus influenzae*), a 10-month old girl was admitted to a hospital based in Genoa (Italy) for the surgical management of grade 4 subglottic stenosis. During hospitalization, a KPC-producing *Klebsiella pneumoniae* strain (KPC-*Kp*) was detected from the tracheal aspirate and urine samples. In October 2020, the patient was discharged at home. One month later, the patient was admitted to the Alessandro Manzoni Hospital (Lecco, Italy) due to respiratory distress. At admission, the patient underwent a screening rectal swab for multidrug-resistant organisms that was directly streaked on different selective media including both a specific chromogenic medium for carbapenem-resistant Enterobacterales (*Brilliance* CRE Agar, Oxoid) and a MacConkey agar plate (bioMérieux) where disks of ertapenem (10 μg) and meropenem (10 μg) were placed. As a result, two carbapenem-resistant strains were routinely identified using VITEK 2 (bioMérieux) and MALDI-TOF MS (VITEK MS, bioMérieux; software version, v3.2 Database): *Escherichia coli* LC-1302–2020 (confidence value, 99.9%) and *K. oxytoca* LC-1303–2020 (confidence value, 99.9%). Notably, strain LC-1303–2020 was also identified as *K. oxytoca* (score 2.28) by using another MALDI-TOF MS apparatus [Bruker; FlexControl v3.4 (build 135); MBT Compass v4.1.100.10; BDAL RUO Library 10 (9607 MSPs)]. The infant was discharged after 2 weeks of hospitalization, where no infections due to carbapenem-resistant Enterobacterales were recorded.

Based on whole-genome sequencing (WGS) data and the Type (Strain) Genome Server (https://tygs.dsmz.de/), the *E. coli* species identification was confirmed, whereas *K. oxytoca* was actually a *K. grimontii*. Antimicrobial susceptibility testing performed using a broth microdilution GNX2F Sensititre panel (Thermo Fisher Scientific) indicated that both isolates were resistant to different classes of antibiotics and showed reduced susceptibility to carbapenems (Table S1).

WGS was performed combining NovaSeq 6000 (Illumina) and MinION (SQK-RBK004 library; FLO-MIN 106D R9 flow-cell; Oxford Nanopore Technologies) to generate complete genome assemblies (i.e., circular) with Unicycler v0.4.8 using the hybrid pipeline with default parameters as previously described [2, 5, 6]. The complete hybrid genomes were analyzed with the tools from the Center for Genomic Epidemiology (www.genomicepidemiology.org/). The genome assemblies of LC-1302–2020 (GenBank: CP091756-CP091761) and LC-1303–2020 (GenBank: CP091752-CP091755) are available under BioProject PRJNA801146.

*E. coli* LC-1302–2020 belonged to sequence type 10 (ST10) and its chromosome harbored the *mdfA* ARG. The strain also carried 5 plasmids, of which p1-LC-1302–2020-KPC3 (298.9 kb) of IncFIB(pQil) replicon sequence (also known as pQil) and possessing *bla*_KPC-3_, *bla*_CTX-M-15_, *bla*_TEM-1B_, Δ*bla*_OXA-1-like_, *aac(3)-IIa*, *aac(6′)-Ib-cr*, *aph(3″)-Ib*, *aph(6)-Id*, *dfrA14*, *qnrB1*, *sul2*, and *tet(A)* ARGs (Fig. 1). *K. grimontii* LC-1303–2020 belonged to a new ST (ST391) since it carried a new *infB* allele (*infB*-54). Its chromosome harbored the *bla*_OXY-6–4_ gene. Three plasmids were also present, of which plasmid p1-LC-1303–2020-KPC3 (252.9 kb) carried an identical replicon sequence and ARGs as in p1-LC-1302–2020-KPC3 (Fig. 1).

**Fig. 1:**
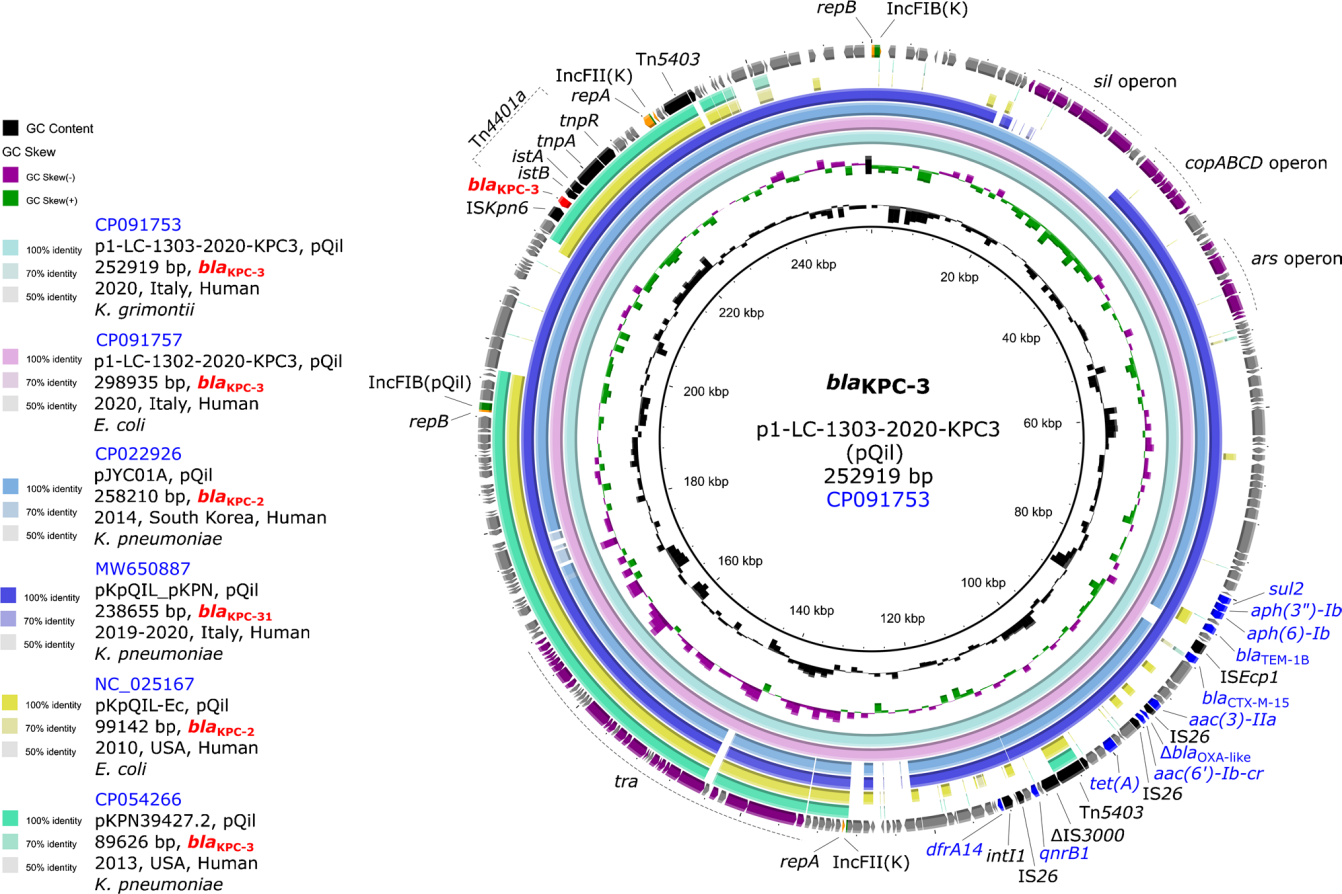
Circular BLASTn comparison of the *bla*_KPC-3_-carrying plasmid in LC-1302–2020 and LC-1303–2002 against other deposited plasmids. Plasmids and their similarities are represented by the colored rings. The CDS/genes and IS elements of interest are represented by colored arrows (red: *bla*_KPC-3_; blue: other ARGs; black: IS elements; orange: replicon genes; green: replicon sequence type) with corresponding annotations (red: *bla*_KPC-3_; blue: other ARGs). The approximate regions for the *sil*, *copACBD*, and *ars* operons, as well as *tra* genes, are indicated by the dashed lines and purple CDS/genes. The approximate region of the transposon associated with *bla*_KPC-3_ (Tn*4401a*) is shown above with dashed lines. For the plasmid comparison, we show the carbapenemase gene of the reference plasmid (in black), name, main replicon sequence type, size, and the GenBank accession (in blue); on the left, we show the GC content, GC skew, and the metadata corresponding to the plasmids used for the circular BLASTn comparison (GenBank accession [in blue], plasmid name, main replicon sequence type, size, *bla*_KPC_ [in red], isolation year, country, isolation source, and host. The IS annotations shown were annotated with ISfinder (https://www-is.biotoul.fr/) using BLASTx search. The circular BLASTn comparison was generated with BLAST Ring Image Generator v0.95 (https://github.com/happykhan/BRIG)

Both plasmids were identical to each other (identity, 99.97%) and harbored the *bla*_KPC-3_ in the archetypal Tn*4401a* element [7]. However, p1-LC-1302–2020-KPC3 was ~ 40 kb larger, possibly due to a duplication event (Fig. S1). Similar duplications have been reported in other *bla*_KPC-3_-carrying pQil (*bla*_KPC-3_-pQil) plasmids in *K. pneumoniae* isolated from the same patient [8].

More importantly, both plasmids were closely related (coverage: 92–96%; identity: 99.25–100%; PLSDB Mash distribution plasmid search analysis) to two other deposited pQil plasmids hosted in *K. pneumoniae*: a *bla*_KPC-2_ plasmid (pJYC01A) from an outbreak in South Korea and a *bla*_KPC-31_ plasmid (pKpQIL_pKPN) recently isolated during a study in Italy (Fig. 1) [9, 10]. In this latter survey, it was also noted a high prevalence of high-risk ST512 KPC-*Kp* strains that possessed the pQil plasmid, suggesting the endemicity of this MGE [9]. Overall, these observations may indicate that *K. grimontii* cooperates with *K. pneumoniae* in the dissemination of such hyperepidemic multidrug resistance plasmids. It can be also speculated that the KPC-*Kp* strain colonizing the intestinal tract of the infant during the first hospitalization was the donor of the *bla*_KPC-3_-pQil plasmid to either *E. coli* LC-1302–2020 or *K. grimontii* LC-1303–2020. Unfortunately, such KPC-*Kp* strain was not available for further WGS analyses and plasmid-to-plasmid comparison.

To support our hypotheses, liquid conjugation experiments with the rifampicin-resistant *E.coli* recipient strain J53d-R1 were conducted at 37 °C for 16 h as previously done [2]. Transconjugants (TCs) were selected on MacConkey agar plates supplemented with rifampicin (50 mg/L) and ampicillin (100 mg/L). TCs showing reduced susceptibility to β-lactams and other classes of antibiotics were obtained (Table S1) with both donor strains. In particular, the conjugation efficiencies (average of 3 replicates) were: 1.2 × 10^−4^ for *E. coli* LC-1302–2020 and 1.8 × 10^−7^ for *K. grimontii* LC-1303–2020. The obtained TCs were *bla*_KPC_-positive according to a PCR performed as previously done [11]. These results confirm the ability of *K. grimontii* to transfer the *bla*_KPC-3_-pQil plasmid to other Enterobacterales, such as *E. coli*.

To further investigate the spread of the *bla*_KPC_-possessing *K. grimontii* (KPC-*Kg*) strains, a database search for other genomes (File S1) and core genome alignment were conducted as previously done (35′965 SNVs across 12 genomes; 88.1% average alignment) [2, 5, 12, 13]. As shown in Fig. 2, we further identified 3 *bla*_KPC-3_- and 8 *bla*_KPC-2_-positive genomes (mostly from North America) belonging to distinct STs. As expected, *K. grimontii* strain LC-1303–2020 was unique from all other KPC-*Kg* (range of ∆SNVs: 14′153–14′712). As well, read mapping of all other KPC-*Kg* against p1-LC-1303–2020-KPC3 confirmed that this pQil plasmid was not present in any of those 11 genomes (data not shown). Notably, as we have shown in our previous work exploring the spread of *bla*_VIM-1_-possessing *K. grimontii*, more KPC-*Kg* (mostly misidentified as *K. oxytoca*) in human and environmental sources have been identified since [2].

**Fig. 2:**
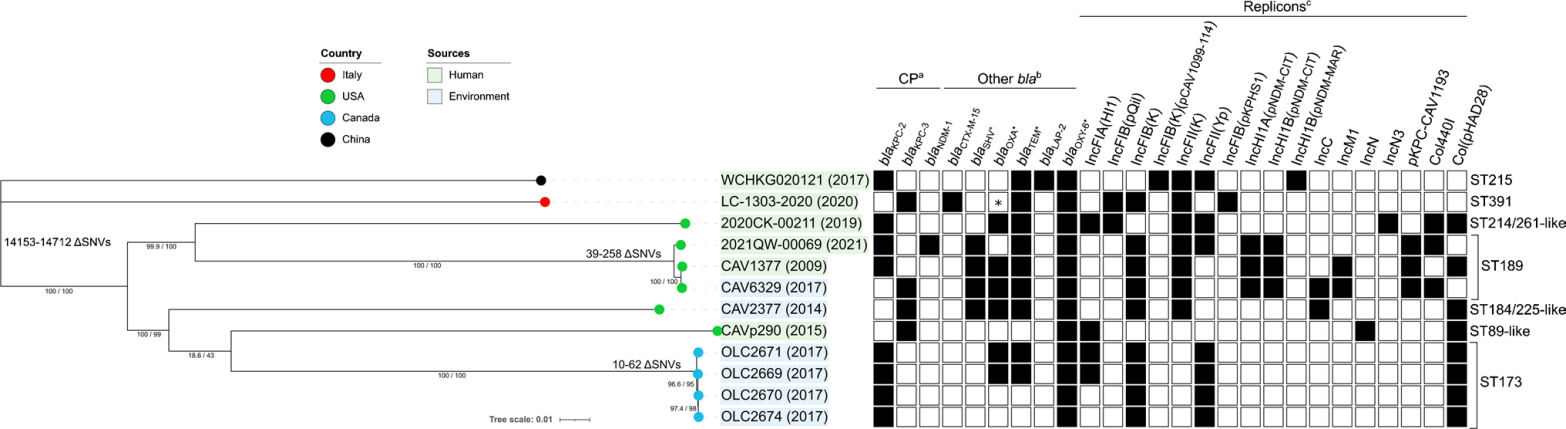
Core genome phylogeny of LC-1303–2020 and other *bla*_KPC-2/-3_-carrying *K. grimontii* (*n* = 12). A total of 8 *K. grimontii* genomes included from publicly available databases (retrieval date: 16–18/Feb/2022; NCBI Genomes, *n* = 143; Pathogen Watch, *n* = 99) were screened for *bla*_KPC_ and *bla*_OXY-6_ genes with Kleborate v2.0.4 with default parameters. Sequence type was determined with MLST v2.19.0 using the *K. oxytoca s*cheme. Simultaneously, the NCBI Pathogen Detection web tool (https://www.ncbi.nlm.nih.gov/pathogens) was used to identify *K. grimontii* genomes deposited under the *K. oxytoca* organism group (*n* = 1000; query: “taxgroup_name:Klebsiella oxytoca AND AMR_genotypes:blaOXY-6* AND AMR_genotypes:blaKPC*”), which resulted in 3 nonredundant *bla*_KPC_- and *bla*_OXY-6_-positive *K. grimontii* genomes. Genomes with no BioSample metadata were excluded (*n* = 30). Genome assemblies were generated with SPAdes v3.14.0 with read correction (parameter: careful) and used for final species ID with TYGS, ARG and replicon sequence screening with the CGE tools (ResFinder v4.1; PlasmidFinder v2.1). A recombination-free core genome alignment was conducted with Snippy v4.4.5 and Gubbins v2.3.4 with default parameters using the complete genome of LC-1303–2020 as reference. Phylogeny was inferred by maximum likelihood with IQ-TREE v2.1.2 using a GTR nucleotide substitution model with ascertainment bias correction (parameter: GTR + ASC) and 1000 ultrafast bootstrap (UFBoot) (parameter: -bb) and the SH-aLRT test (parameter: -alrt). The tree was visualized and annotated with iTOL v1.6. Countries are represented by the colored circles; strain or isolate name and collection date are highlighted by isolation source (as per BioSample metadata). Delta SNVs (∆SNVs) represent core genome similarities between two or more genomes. Bootstrap support values are shown on branches (SH-aLRT and UFboot, respectively). The tree scale represents the average number of nucleotide substitutions per site. ^a^Carbapenemase gene. ^b^Other *bla* genes present; an asterisk corresponds to a variant from the same family. An asterisk in *bla*_OXA_ in LC-1303–2020 corresponds to Δ*bla*_OXA-1-like_. ^c^Replicon sequence types identified by PlasmidFinder at 50% minimum identity.

The identification of pQil replicon sequences in other deposited *K. grimontii* (Fig. 2) suggests an exchange, so far undetected, of this type of plasmids between closely related species (e.g., *K. pneumoniae* to *K. grimontii*). We also note that *bla*_KPC_-pQil plasmids have been reported worldwide in other species (e.g., *E. coli* and *Klebsiella aerogenes*) [7, 14]. Our conjugation experiment results and the finding of *E. coli* LC-1302–2020 demonstrated, in fact, that the horizontal transfer of the *bla*_KPC-3_-pQil plasmid between different species is possible and can favor the expansion of KPC-producing pathogens.

In conclusion, we reported the first *bla*_KPC-3_-carrying *K. grimontii* isolate. The strain was isolated from the gut of a patient concurrently with an *E. coli* carrying the same *bla*_KPC-3_-pQil conjugative plasmid. We also showed that other non-clonally related KPC-*Kg* possessing *bla*_KPC-2/-3_ were published and/or erroneously deposited in various databases as *K. oxytoca* [15].

Overall, our findings emphasize the importance of correctly identifying *K. grimontii* because it represents an emerging reservoir of ARGs threatening our antibiotic armamentarium. As long as MALDI-TOF MS databases are not updated to correctly identify this pathogen, we recommend achieving species identification by using molecular methods (e.g., sequencing of *bla*_OXY_) or, alternatively, reporting the results as *K. oxytoca* complex [3].

## Supplementary Information

Below is the link to the electronic supplementary material.

Supplementary file1 (DOCX 21 KB)

Supplementary file2 (PPTX 397 KB)

Supplementary file3 (XLSX 13 KB)

## Data Availability

The genome assemblies of strains LC-1302–2020 (GenBank: CP091756-CP091761) and LC-1303–2020 (GenBank: CP091752-CP091755) are available under BioProject PRJNA801146.

## References

[1] Passet V, Brisse S (2018) Description of *Klebsiella grimontii* sp. nov. Int J Syst Evol Microbiol 68:377–381 10.1099/ijsem.0.002517 29205126

[2] Campos-Madueno EI, Moser AI, Risch M, Bodmer T, Endimiani A (2021). Exploring the global spread of *Klebsiella grimontii* isolates possessing *bla*_VIM-1_ and *mcr-9*. Antimicrob Agents Chemother.

[3] Yang J, Long H, Hu Y, Feng Y, McNally A, Zong Z (2022). *Klebsiella oxytoca* complex: update on taxonomy, antimicrobial resistance, and virulence. Clin Microbiol Rev.

[4] Liu L, Feng Y, Hu Y, Kang M, Xie Y, Zong Z (2018). *Klebsiella grimontii*, a new species acquired carbapenem resistance. Front Microbiol.

[5] Campos-Madueno EI, Moser AI, Jost G, Maffioli C, Bodmer T, Perreten V (2022). Carbapenemase-producing *Klebsiella pneumoniae* strains in Switzerland: human and non-human settings may share high-risk clones. J Glob Antimicrob Resist.

[6] Moser AI, Campos-Madueno EI, Sendi P, Perreten V, Keller PM, Ramette A (2021). Repatriation of a patient with COVID-19 contributed to the importation of an emerging carbapenemase producer. J Glob Antimicrob Resist.

[7] Pitout JD, Nordmann P, Poirel L (2015). Carbapenemase-producing *Klebsiella pneumoniae*, a key pathogen set for global nosocomial dominance. Antimicrob Agents Chemother.

[8] Stohr J, Verweij JJ, Buiting AGM, Rossen JWA, Kluytmans J (2020). Within-patient plasmid dynamics in *Klebsiella pneumoniae* during an outbreak of a carbapenemase-producing *Klebsiella pneumoniae*. PLoS One.

[9] Carattoli A, Arcari G, Bibbolino G, Sacco F, Tomolillo D, Di Lella FM (2021). Evolutionary trajectories toward ceftazidime-avibactam resistance in *Klebsiella pneumoniae* clinical isolates. Antimicrob Agents Chemother.

[10] Song JE, Jeong H, Lim YS, Ha EJ, Jung IY, Jeong W (2019). An outbreak of KPC-producing *Klebsiella pneumoniae* linked with an index case of community-acquired KPC-producing isolate: epidemiological investigation and whole genome sequencing analysis. Microb Drug Resist.

[11] Endimiani A, Carias LL, Hujer AM, Bethel CR, Hujer KM, Perez F (2008). Presence of plasmid-mediated quinolone resistance in *Klebsiella pneumoniae* isolates possessing *bla*_KPC_ in the United States. Antimicrob Agents Chemother.

[12] Brilhante M, GobeliBrawand S, Endimiani A, Rohrbach H, Kittl S, Willi B (2021). Two high-risk clones of carbapenemase-producing *Klebsiella pneumoniae* that cause infections in pets and are present in the environment of a veterinary referral hospital. J Antimicrob Chemother.

[13] Campos-Madueno EI, Bernasconi OJ, Moser AI, Keller PM, Luzzaro F, Maffioli C et al (2020) Rapid increase of CTX-M-producing *Shigella sonnei* isolates in Switzerland due to spread of common plasmids and international clones. Antimicrob Agents Chemother 64:e01057–20 10.1128/AAC.01057-20 PMC7508577 32718957

[14] Chen L, Chavda KD, Melano RG, Jacobs MR, Koll B, Hong T (2014). Comparative genomic analysis of KPC-encoding pKpQIL-like plasmids and their distribution in New Jersey and New York Hospitals. Antimicrob Agents Chemother.

[15] Cooper A, Carter C, McLeod H, Wright M, Sritharan P, Tamber S (2021). Detection of carbapenem-resistance genes in bacteria isolated from wastewater in Ontario. FACETS.

