# Supplementary material for: Simultaneous gut colonization by Klebsiella grimontii and Escherichia coli co-possessing the blaKPC-3-carrying pQil plasmid
Source: Eur J Clin Microbiol Infect Dis. 2022 May 28;41(7):1087–91. doi: 10.1007/s10096-022-04462-z (PMC9250482; doi:10.1007/s10096-022-04462-z)
Supplement: Supplementary file 1 — Supplementary file1 (DOCX 21 KB) [file 10096_2022_4462_MOESM1_ESM.docx]

Supplementary Table S1. Phenotypic characterization of the KPC-3-producing *E. coli* and *K. grimontii* strains and corresponding *E. coli* J53d-R1 transconjugants (TC)

| **Antibiotics** | MIC values (mg/L) ^a^ | | | | |
| --- | --- | --- | --- | --- | --- |
|  | *E. coli*  LC-1302-2020 | *K. grimontii*  LC-1303-2020 | *E. coli* J53d-R1  LC-1302-2020-TC | *E. coli* J53d-R1  LC-1303-2020-TC | *E. coli*  J53d-R1 |
| Piperacillin-tazobactam | >64/4 (R) | >64/4 (R) | >64/4 (R) | >64/4 (R) | ≤8/4 (S) |
| Ticarcillin-clavulanate | >128/2 (R) | >128/2 (R) | >128/2 (R) | >128/2 (R) | ≤16/2 (S) |
| Cefotaxime | >32 (R) | >32 (R) | 8 (R) | 8 (R) | ≤1 (S) |
| Ceftazidime | >16 (R) | >16 (R) | >16 (R) | >16 (R) | ≤1 (S) |
| Cefepime | 16 (R) | 8 (R) | 4 (I) | 4 (I) | ≤1 (S) |
| Aztreonam | >16 (R) | >16 (R) | >16 (R) | >16 (R) | ≤2 (S) |
| Imipenem | ≤1 (S) | 4 (I) | 2 (S) | ≤1 (S) | ≤1 (S) |
| Meropenem | ≤1 (S) | 8 (I) | ≤1 (S) | ≤1 (S) | ≤1 (S) |
| Doripenem | 1 (S) | 1 (S) | 0.5 (S) | 0.5 (S) | ≤0.12 (S) |
| Ertapenem | 2 (R) | 4 (R) | 0.5 (S) | 1 (R) | ≤0.25 (S) |
| Gentamicin | >8 (R) | >8 (R) | ≤1 (S) | 2 (S) | ≤1 (S) |
| Tobramycin | >8 (R) | >8 (R) | ≤1 (S) | 2 (S) | ≤1 (S) |
| Amikacin | ≤4 (S) | ≤4 (S) | ≤4 (S) | 8 (S) | ≤4 (S) |
| Levofloxacin | ≤1 (S) | ≤1 (S) | ≤1 (S) | ≤1 (S) | ≤1 (S) |
| Ciprofloxacin | 1 (R) | 1 (R) | ≤0.25 (S) | ≤0.25 (S) | ≤0.25 (S) |
| Doxycycline | 16 (NA) | 16 (NA) | ≤2 (NA) | 4 (NA) | ≤2 (NA) |
| Minocycline | ≤2 (NA) | 4 (NA) | ≤2 (NA) | 4 (NA) | ≤2 (NA) |
| Tigecycline | ≤0.25 (S) | 0.5 (NA) | ≤0.25 (S) | ≤0.25 (S) | ≤0.25 (S) |
| Trimethoprim/sulfamethoxazole | >4/76 (R) | >4/76 (R) | ≤0.5/9.5 (S) | ≤0.5/9.5 (S) | ≤0.5/9.5 (S) |
| Colistin | ≤0.25 (S) | ≤0.25 (S) | ≤0.25 (S) | ≤0.25 (S) | ≤0.25 (S) |
| Polymyxin B | ≤0.25 (NA) | ≤0.25 (NA) | ≤0.25 (NA) | ≤0.25 (NA) | ≤0.25 (NA) |

**Note.** R, resistant; I, susceptible, increased exposure; S, susceptible; NA, not available; -, not tested

**^a^** MICs were obtained with microdilution Sensititre panel GNX2F and interpreted according to the EUCAST 2021 criteria (version 11.0)
