# Supplementary material for: Simultaneous gut colonization by Klebsiella grimontii and Escherichia coli co-possessing the blaKPC-3-carrying pQil plasmid
Source: Eur J Clin Microbiol Infect Dis. 2022 May 28;41(7):1087–91. doi: 10.1007/s10096-022-04462-z (PMC9250482; doi:10.1007/s10096-022-04462-z)
Supplement: Supplementary file 2 — Supplementary file2 (PPTX 397 KB) [file 10096_2022_4462_MOESM2_ESM.pptx]

## Slide 1
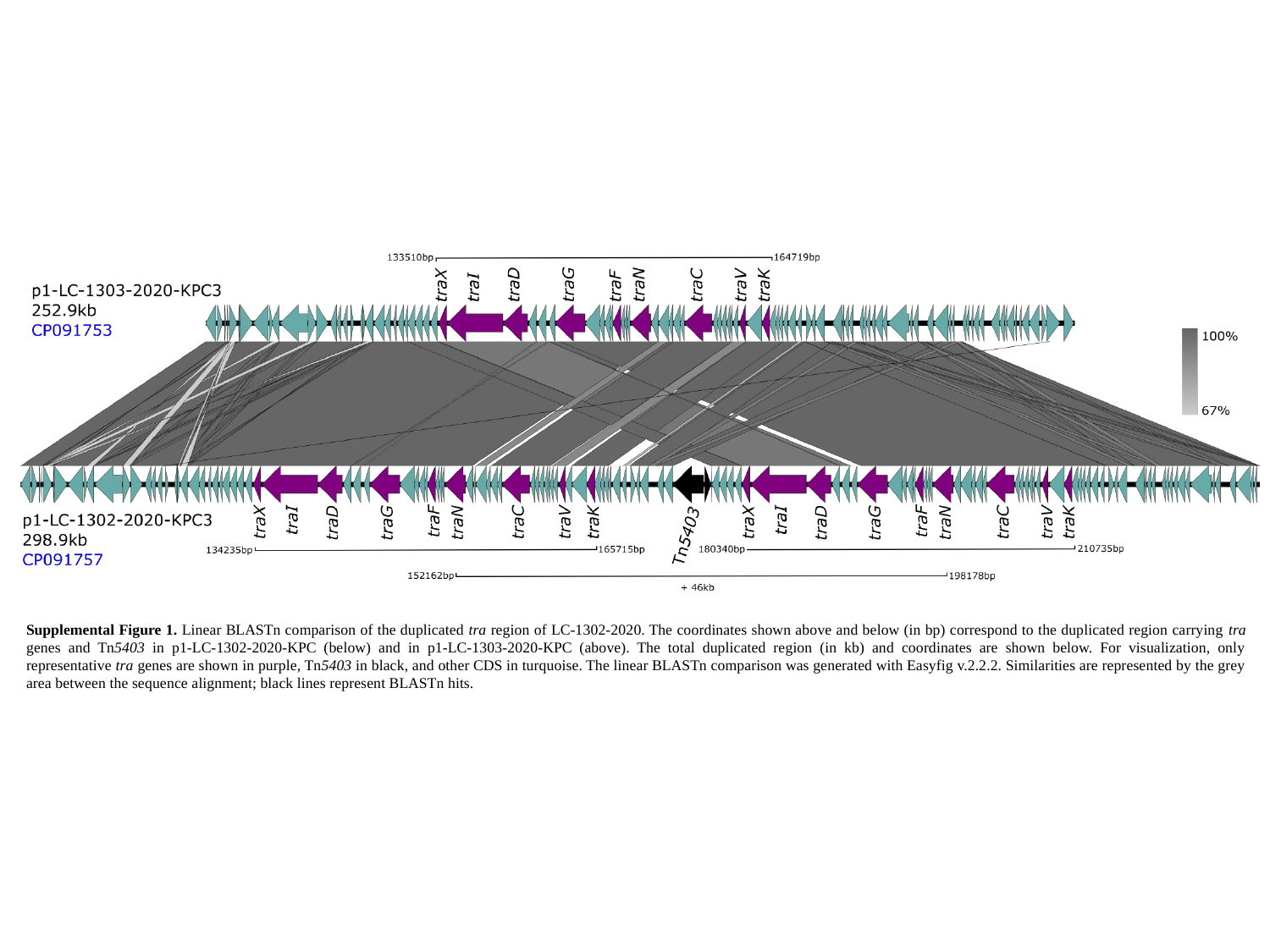

Supplemental Figure 1. Linear BLASTn comparison of the duplicated tra region of LC-1302-2020. The coordinates shown above and below (in bp) correspond to the duplicated region carrying tra genes and Tn5403 in p1-LC-1302-2020-KPC (below) and in p1-LC-1303-2020-KPC (above). The total duplicated region (in kb) and coordinates are shown below. For visualization, only representative tra genes are shown in purple, Tn5403 in black, and other CDS in turquoise. The linear BLASTn comparison was generated with Easyfig v.2.2.2. Similarities are represented by the grey area between the sequence alignment; black lines represent BLASTn hits.
